# Synthetic datasets for open software development in rare disease research

**DOI:** 10.1186/s13023-024-03254-2

**Published:** 2024-07-15

**Authors:** Ibraheem Al-Dhamari, Hammam Abu Attieh, Fabian Prasser

**Affiliations:** 1 Medical Informatics Group, Berlin Institute of Health at Charité - Universitätsmedizin, Berlin, Germany; 2 Institute of Software Technology (IST), Koblenz University, Koblenz, Germany

**Keywords:** Synthetic data, Rare diseases, Development, Evaluation, Statistics

## Abstract

**Background:**

Globally, researchers are working on projects aiming to enhance the availability of data for rare disease research. While data sharing remains critical, developing suitable methods is challenging due to the specific sensitivity and uniqueness of rare disease data. This creates a dilemma, as there is a lack of both methods and necessary data to create appropriate approaches initially. This work contributes to bridging this gap by providing synthetic datasets that can form the foundation for such developments.

**Methods:**

Using a hierarchical data generation approach parameterised with publicly available statistics, we generated datasets reflecting a random sample of rare disease patients from the United States (US) population. General demographics were obtained from the US Census Bureau, while information on disease prevalence, initial diagnosis, survival rates as well as race and sex ratios were obtained from the information provided by the US Centers for Disease Control and Prevention as well as the scientific literature. The software, which we have named SynthMD, was implemented in Python as open source using libraries such as Faker for generating individual data points.

**Results:**

We generated three datasets focusing on three specific rare diseases with broad impact on US citizens, as well as differences in affected genders and racial groups: Sickle Cell Disease, Cystic Fibrosis, and Duchenne Muscular Dystrophy. We present the statistics used to generate the datasets and study the statistical properties of output data. The datasets, as well as the code used to generate them, are available as Open Data and Open Source Software.

**Conclusion:**

The results of our work can serve as a starting point for researchers and developers working on methods and platforms that aim to improve the availability of rare disease data. Potential applications include using the datasets for testing purposes during the implementation of information systems or tailored privacy-enhancing technologies.

## Background

Despite their individual rarity (e.g. defined as 1 in 1,700 in the United States (US) [1] and 1 in 2,000 in Europe [2]), rare diseases collectively affect a large population and often manifest as chronic and life-threatening conditions [3].

The availability of large enough datasets on affected patients is important for developing new diagnostics and therapy options and for applying modern data science and artificial intelligence techniques. Given the rarity of such diseases, scientific collaboration and data sharing are important to achieve this [4].

However, sharing rare disease data raises questions related to patient privacy, as its disclosure may lead to societal stigma, discrimination, or harassment [5]. Moreover, the privacy of rare disease patients is particularly challenging to protect, because their diseases affect only a small demographic, increasing the risk of privacy breaches. This calls for specialized privacy-enhancing technologies tailored to the needs of rare disease research. This need results in a dilemma. New and tailored privacy protection methods must be developed, but as the data on which they are being developed must stay confidential, open science practices, external evaluations, and transparent method development are severely limited.

Recently, machine-learning (ML)-based synthetic data generation methods have been promoted as a versatile tool for sharing data while preserving privacy. The general idea is to use ML models trained on sensitive data to generate data that mirrors important statistical properties while not containing any real-world personal information [6–8]. However, the generation of synthetic data requires trading off the degree to which statistical properties are preserved with the degree of privacy protection achieved [9], they struggle with longitudinal data [10] and there is yet no generally accepted technique that could be applied to rare diseases datasets.

In the work described in this article, we took a step back and generated synthetic rare disease datasets from publicly available statistical information. While these datasets are not suitable for generating new insights into rare diseases, they can be utilized for the development and evaluation of software for rare disease research.

For example, the datasets could be used to develop tailored synthesis or anonymisation mechanisms and to publish them along with open data on their evaluation. Moreover, the datasets could serve as test datasets in the development of information systems, such as rare disease biobank information systems and registries [11], that reflect the expected statistical properties.

## Methods

### Tool selection

As already mentioned, the wide range of ML-based synthetisation methods and libraries available are not suited for the type of synthetisation process which we aimed to perform. Before implementing the method described in the remainder of this section, we therefore screened the landscape of available modelling-based data generation tools. *Faker* is a popular Python package for creating synthetic data for software development and testing purposes, but it has not been designed to use models of dependencies between variables or complex statistical properties of real-world data [12]. The *Synthetic Data Vault* is a comprehensive tool for data generation based on information learned from a given database [13], which does not suit our application scenario. *Synner* [14] is an interesting tool supporting data generation processes that are very close to what we planned to do, but it focuses on interactions through a comprehensive user interface, while we were interested in a scripted approach. The *synthpop* package for the R statistical computing environment follows a modelling approach rather than a machine learning approach, but it has been designed to generate synthetic data from an individual-level input dataset from which the models are extracted [15]. *Synthea* is probably the most well-known tool for generating synthetic patient trajectories out of statistical information [16]. However, Synthea is complex to configure and it has not been specifically engineered to produce data for a single disease across various regions but is more focused on generating diverse patient population. Moreover, the simulation-based approach of Synthea makes it relatively slow (generation of approximately 1000 patient records per minute in test performed). We hence decided to implement SynthMD, a small and lightweight library tailored to generating datasets following the statistical distributions and properties outlined in the following sections.

### Statistics collection

**Fig. 1:**
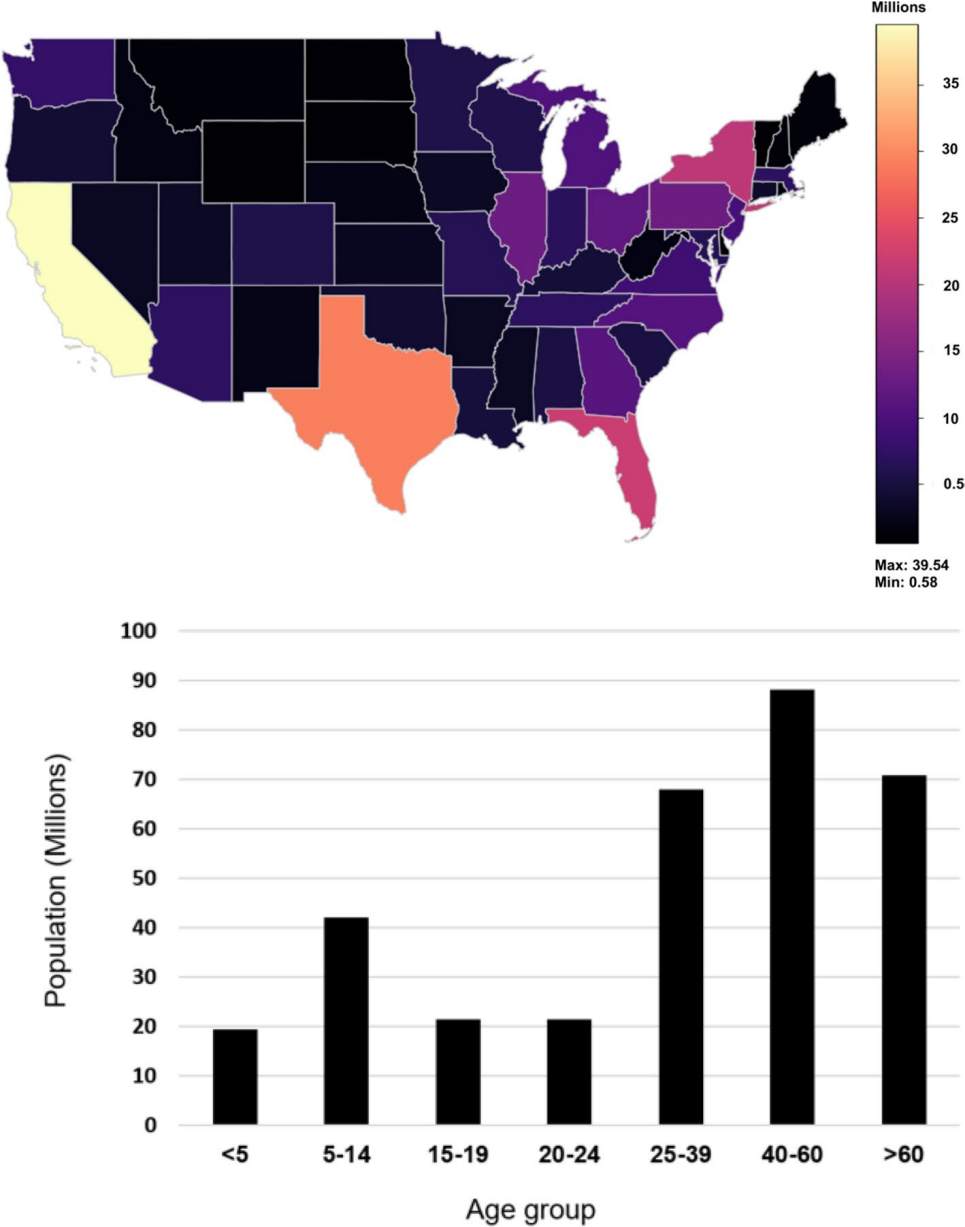
Charts illustrating the basic demographic data collected about the US population (year: 2020): **a** Population per state, **b** Population per age is categorized by different age groups for simplicity

We decided to generate synthetic data modelling the US population, as a lot of statistical information is available for US citizens and the population is quite diverse. We hence collected general population demographics as well as disease-specific statistics. Population statistics in regards to gender, race, and age from each US state, Washington DC is included, were collected from the US Census Bureau using their official Application Programming Interface (API). The data collected also included age statistics from ages 0 to 84 years, with all ages from 85 years and onwards being grouped together. An overview is provided in Fig. 1.

We focused on three specific rare diseases due to their broad impact on US residents, as well as their differences concerning gender and racial groups: Sickle Cell Disease (SCD; ORPHA code: 232), Cystic Fibrosis (CF; ORPHA code: 586), and Duchenne Muscular Dystrophy (DMD; ORPHA code: 98896). Disease statistics, such as prevalence, survival rates for various age groups, race and sex ratios, and clinical parameters, were collected from both academic literature and official resources. An overview is provided in Tables 1, 2 and 3.

**Table 1:** Harmonized collected statistics about sickle cell disease

Category	Variable	Value	Distribution
General	Prevalence	–	1/3300
Demographics	Race	African-American	73.10%
		European-American	3.00%
		Others	23.90%
Demographics	Sex	Male	50.00%
		Female	50.00%
Clinical course	Initial diagnosis	Months after birth	5 - 6, $\mu = 5.5 \pm 0.5$
Clinical course	Death rate per 100,000	<5 years old	0.47
		5-14 years old	0.30
		15-19 years old	0.70
		20-24 years old	1.35
		25-39 years old	2.75
		40-60 years old	2.85
		>60 years old	1.99
Clinical course	Complete blood count	–	6 - 11 g/dL, $\mu = 8.5 \pm 2.5$
	Reticulocyte count		2.0 - 3.0%, $\mu = 2.5 \pm 0.5$

**Table 2:** Harmonized collected statistics about cystic fibrosis

Category	Variable	Value	Distribution
General	Prevalence	–	1/10311
Demographics	Race	African-American	3.50%
		European-American	91.40%
		Others	5.10%
Demographics	Sex	Male	51.70%
		Female	49.30%
Clinical course	Initial diagnosis	Days after birth	2 - 3, $\mu = 2.5 \pm 0.5$
Clinical course	Death rate per 100,000	<5 years old	6.23
		5-14 years old	12.46
		15-19 years old	40.50
		20-24 years old	71.65
		25-39 years old	280.37
		40-60 years old	190.03
		>60 years old	121.50
Clinical course	Chloride level	–	30.0 - 118.6 mmol/L
			$$\mu = 74.3 \pm 44.3$$

**Table 3:** Harmonized collected statistics about duchenne muscular dystrophy

Category	Variable	Value	Distribution
General	Prevalence	–	1/6000
Demographics	Race	African-American	29.00%
		European-American	43.00%
		Others	28.00%
Demographics	Sex	Male	99.99%
		Female	0.01%
Clinical parameters	Initial diagnosis	Years after birth	1 - 3, $\mu = 2 \pm 1$
Clinical parameters	Death rate per 100,000	<5 years old	200
		5-14 years old	200
		15-19 years old	200
		20-24 years old	40,500
		25-39 years old	73,900
		40-60 years old	86,700
		>60 years old	99,990
Clinical parameters	Creatine kinase level (CK)	–	350 - 23,200 units/L
			$$\mu = 11775 \pm 6475$$

Table 1 shows the statistics collected about SCD, which is one of the most common rare diseases, affecting at least 3 million people worldwide, with 100,000 patients in the US alone. The general prevalence of the disease is about 1 in 3300 individuals [17, 18]. Diagnosis of SCD typically includes a complete blood count (CBC), because individuals with SCD usually have fewer red blood cells than normal. Sickle-shaped red blood cells do not circulate as long as normal ones, leading to lower hemoglobin levels between 6 to 11 g/dL. The count of reticulocytes cells (RC), immature red blood cells formed in the bone marrow, tends to be higher in individuals with SCD, often 2 to 3 percent or more [19]. Treatment options are limited, encompassing infection prophylaxis, hydroxyurea, blood transfusion, analgesia, and haematopoietic stem cell transplantation. Newer treatments like gene therapy also exist [17]. In the US, SCD affects roughly 1 out of every 365 Black or African-American births [18, 20].

Table 2 shows that statistics collected about CF, which is a progressive disease that primarily affects the body’s mucus glands, impacting primarily the respiratory and digestive systems in children and young adults [21]. Previously considered the most common life-threatening inherited rare disease in Caucasian children, with a prevalence of 1 in 2500, advances in treatments and disease management have changed the CF population’s characteristics [21, 22]. Disease diagnosis often begins with newborn screening 2 to 3 days after birth, followed by confirmatory tests in the following weeks or months. Common diagnostic tests for CF include the sweat test, which measures sodium and chloride levels, and the Immuno-Reactive Trypsinogen test, analysing trypsinogen, a specific protein found in blood drawn 2 to 3 days after birth [21]. Treatments for CF address both the underlying genetic causes and the symptoms of the disease to improve quality of life [21].

Statistics collected about DMD are illustrated in Table 3. DMD is a rare disease with a prevalence of approximately 1 in 5000 male live births [23]. It is caused by single or multiple exonic deletions or duplications in the dystrophin gene in 80% of cases. The disease gradually weakens and degenerates muscles, particularly skeletal and cardiac muscles. Patients typically become wheelchair dependent around the age of 13, with a mean survival age of 29 years, limited primarily by cardiorespiratory complications [24]. DMD is usually diagnosed within 1-3 years after birth. Creatine kinase is typically elevated in individuals with DMD due to muscle damage and a blood test is often used as an initial diagnostic tool [25]. The majority of patients are male with males constituting 99.99% of diagnosed cases.

### Data preprocessing

The collected statistics underwent preprocessing to generate comparable statistics for all three diseases. We categorised all age values into seven distinct groups: under 5, 5-14, 15-19, 20-24, 25-39, 40-60, and over 60 years old. Another example of such preprocessing is converting all survival rates into the form provided in the tables.

### Data generation

**Fig. 2:**
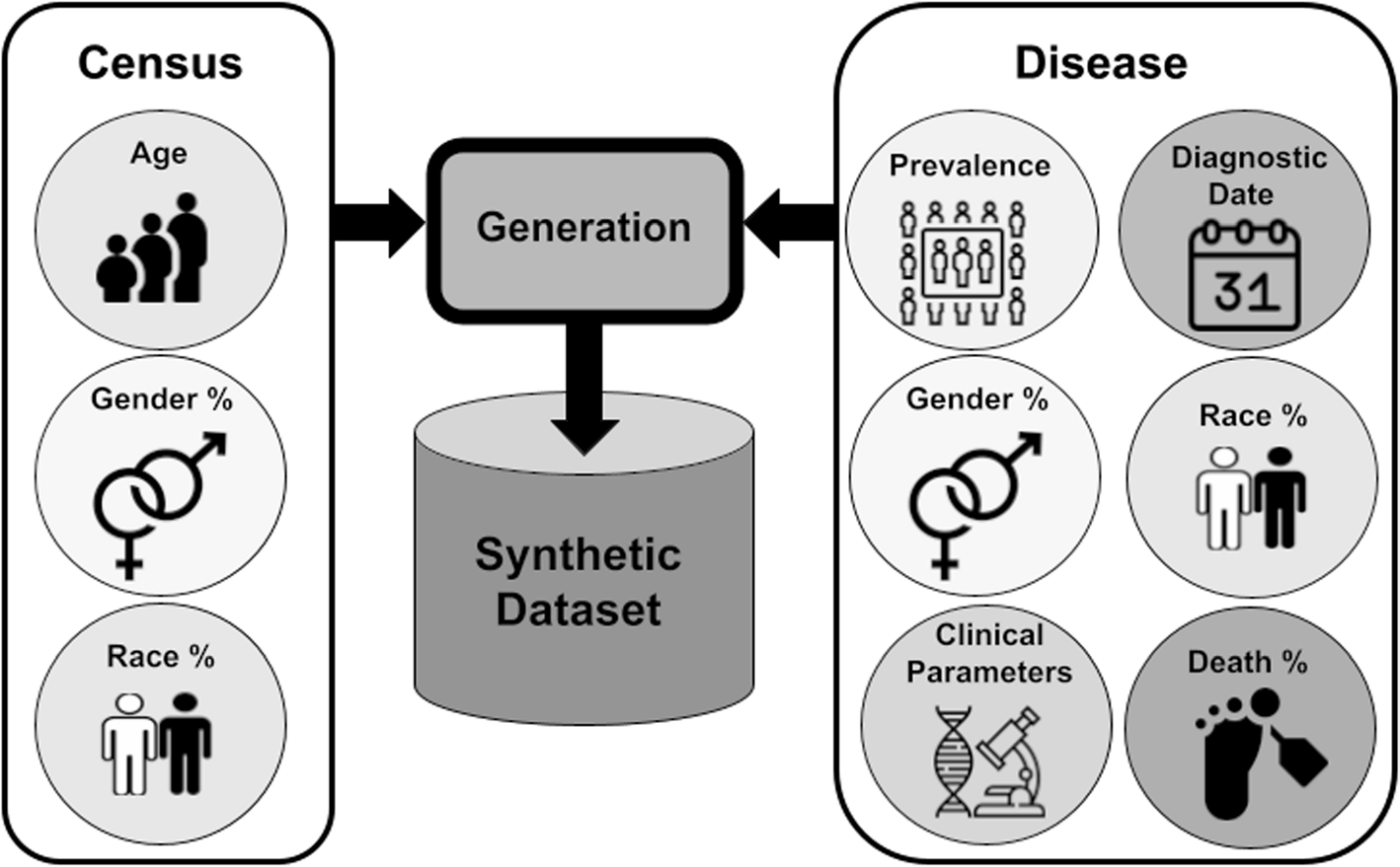
Overview of the synthetic data generation process and the statistics used

An overview of our approach is provided in Fig. 2. The statistical information described in the previous section was encoded in JSON files that were then loaded by a Python script executing the data generation process. Here, we employ a hierarchical approach, characterised by nested loops as shown in Algorithm 1.

In the hierarchical data generation process, the datasets are created on a record-by-record basis. First, the distribution for each variable is obtained. Following this, distributions at subsequent levels are computed, taking into account the values drawn at the preceding levels, leading to the creation of one or multiple records at the terminal level. This approach ensures that interdependencies specified among attributes are captured. A prime example of such a hierarchical relationship is the alignment of city or ZIP code, which is guided by the corresponding state variable drawn at the previous level.

**Figure:**
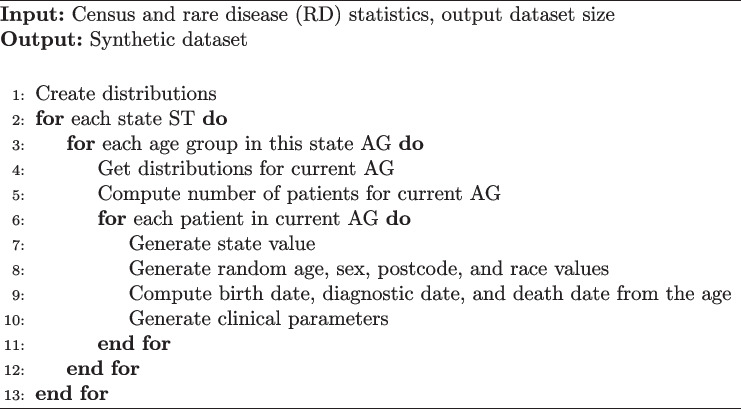
**Algorithm 1** Data generation algorithm

At the root, the algorithm generates a geographic distribution by iterating through the US states. The ZIP code is randomly selected from the set of all ZIP codes associated with the current state. Patient sex is then drawn considering the sex distribution of the age group and within the current state. This distribution takes into account data from both the US census age-sex population statistics and the gender ratio of rare diseases, favouring the latter. The patient’s age is determined through random selection from a drawn age range.

Patient race is selected taking into account both the US Census statistics for the current state and the rare disease race distribution, favouring the latter. Clinical parameters are randomly selected from a normal distribution with the specified parameters.

Finally, the patient’s date of birth is computed from their age, depending on their vital status. For living patients, a random date representative of their age as of 01/01/2023 is generated. The patient’s vital status (dead/alive) is assigned, depending on the patient’s age and the mortality distribution for each age group and state. For deceased patients, their age at the time of death is considered, and a suitable date of birth is generated. The date of diagnosis is generated as specified in the statistics.

## Results

We generated three datasets for the three diseases using SynthMD, capturing all synthetic cases across the US population (about 331 million citizens). Each of the three datasets contains a set of patient records with age (as of 01-01-2023), US state and ZIP code of residence, sex, race, date of birth, date of initial diagnosis as well as potentially a death date. In addition, the files contain one or two clinical parameters.

Table 4 provides an overview of the three generated datasets. It displays the number of male and female patients, the total number of patients, the relative prevalence of the disease within respective populations, and the number of deceased patients. As can be seen, the dataset for SCD contains 100,402 patients, the dataset for CF 32,092 patients and the dataset for DMD 55,218 patients.

**Table 4:** Summary of the three generated datasets

Disease	Male	Female	Total	Prevalence	Deceased
SCD	50,275	50,127	100,402	0.0021	0
CF	16,796	15,296	32,092	0.0007	187
DMD	54,911	307	55,218	0.0012	29,724

Table 5 compares the actual statistics of the generated datasets to the expected statistics presented in the previous section. As can be seen, there are no significant differences between these parameters.

**Table 5:** Actual vs. expected characteristics of the generated datasets

	SCD		CF		DMD	
	Result	Expected	Result	Expected	Result	Expected
Total patients	100,402	100,439	32,092	32,100	55,218	55,242
Deceased patients	0	0	187	191	29,724	29,615
Female [%]	49.92	50.00	47.66	48.30	0.55	0.002
African American [%]	73.20	73.10	4.04	3.50	29.12	29.00
European American [%]	2.96	3.00	91.15	91.40	43.03	43.00
Other American [%]	23.83	23.90	4.80	5.10	27.84	28.00

Table 6 presents an example of how age-dependent statistics are captured in the output data. It lists the size of the underlying population groups, the number of patients as well as the number of deceased patients for CF. As can be seen, the age-related death rates match the ones presented in the previous section. The code and synthetic datasets are publicly available on GitHub^1^.

**Table 6:** Age-dependent statistics of the CF dataset

Age group	Population	Patients	Actual deceased	Expected deceased
<5	19,392,551	1,883	0	0
5-14	42,097,211	4,085	0	0
15-19	21,546,953	2,098	0	0
20-24	21,468,520	2,082	3	2
25-39	67,927,568	6,582	87	82
40-60	88,234,779	8,544	66	72
>60	70,781,388	6,818	31	35

## Discussion

We have generated three synthetic datasets on three different rare diseases using an approach based on publicly available information. The datasets model the complete US population of patients with the respective diseases and samples or subsets can be extracted if smaller datasets or datasets from a specific geographical region are needed. The basic information contained in our synthetic datasets can also be supplemented with further variables, if more comprehensive datasets are required.

A limitation of our tool is that it focuses on tabular data only and cannot be used to generate other critical data types, such as genetic or imaging data. Limitations of our datasets include the fact that their scope is relatively narrow, basically capturing demographics, simple information on disease course and selected diagnosis-relevant clinical parameters only. Moreover, we were not able to retrieve all required statistics from the scientific literature and hence some statistics have been taken from online sources that lack peer review [19]. We also assumed an equal death rate for SCD, despite recent evidence suggesting differences [20]. Finally, we did not consider all potential relationships between the statistics used, such as between race and state of residence.

A noteworthy related work has been presented in [26]. The approach proposed in this paper leverages data augmentation and epidemiological profiles to generate synthetic data for Uveitis, a rare ophthalmological disease. The synthetic data underwent both qualitative evaluation by ophthalmology specialists and quantitative testing using machine learning methods, yielding promising outcomes in regards to data validity.

## Conclusion

In this work, we have presented a simple approach to generating synthetic rare disease datasets for development and evaluation purposes out of publicly available statistics, implemented as a tool called SynthMD. The developments were also driven by our own need for development and evaluation datasets for our research on rare disease-specific anonymisation technologies. By publishing these datasets for other researchers to use in their projects^2^ we hope to contribute to resolving the dilemma around data availability and the need to develop specific privacy-enhancing technologies for sharing rare disease data.

## Data Availability

The code, experimental data, and results supporting the findings of this study are publicly available on GitHub at https://github.com/iaBIH/synth-md.

## Footnotes

1 https://github.com/iaBIH/synth-md

2 https://github.com/iaBIH/synth-md
